# Localisation buccale d’un lymphome de Burkitt: à propos d’un cas

**DOI:** 10.11604/pamj.2017.26.63.5955

**Published:** 2017-02-03

**Authors:** Lamia Kissi, Rajaa El Bouihi, Mouna Lamchahab, Ahmed Alaoui, Ihsane Benyahya

**Affiliations:** 1 Service d’Odontologie Chirurgicale, Centre de Consultations et de Traitements Dentaire de Casablanca, Faculté de Médecine Dentaire de Casablanca, Maroc; 2 Service d’Hématologie, Hôpital 20 Août, Casablanca, Maroc; 3 Laboratoire Anatomo-Pathologie du privé, Maroc

**Keywords:** Lymphome de Burkitt, localisation orale, rôle du médecin dentiste, diagnostic, Burkitt's lymphoma, oral location, role of the dentist, diagnosis

## Abstract

Le lymphome de Burkitt (LB) est une forme de lymphome malin non-Hodgkinien qui provient de l'évolution maligne et de la prolifération de cellules lymphoïdes de type B. Le diagnostic positif repose sur la biopsie d'une masse tumorale ou la ponction de la moelle osseuse révélant la présence de cellules tumorales. Le cas rapporté est celui d'un jeune homme d'une vingtaine d'années qui a été adressé pour des tuméfactions gingivales évoluant depuis 1 mois suite à des extractions dentaires. L'examen anatomopathologique après biopsie complété par l'immunohistochimie conclut à un lymphome de Burkitt. La prise en charge a consisté en une chimiothérapie. Bien que rare, le lymphome de Burkitt est une tumeur agressive qui représente un véritable problème de santé publique d'où la place importante qu'occupe le médecin dentiste dans le diagnostic précoce afin de permettre une prise en charge rapide et appropriée seule garante de la guérison.

## Abstract (en)

Burkitt's lymphoma (LB) is a type of malignant non-Hodgkin's lymphoma originating from malignant B-cell transformation and proliferation. Positive confirmation is based on biopsy of the tumor mass or bone marrow aspiration revealing the presence of tumor cells. We here report the case of a young man, about twenty years old, addressed for post tooth extraction gingival swellings evolving for 1 month. Anatomopathologic examination after biopsy complemented by immunohistochemistry confirmed the diagnosis of Burkitt's lymphoma. Treatment was based on chemotherapy. Although Burkitt's lymphoma is rare, it is an aggressive tumor that represents a real public health problem, hence the role of the dentists in early diagnosis, in order to allow rapid and appropriate management of the disease which is vital to the healing process.

## Introduction

Le lymphome de Burkitt (LB) représente 30 à 40% des lymphomes non hodgkiniens (LNH) de l'enfant [1, 2]. Il représente dans les régions équatoriales 50% des cancers de l'enfant et plus de 70% des LNH [3]. Le pronostic de cette tumeur s'est considérablement amélioré ces dernières années grâce à des protocoles thérapeutiques intensifs et courts notamment dans les pays en voie de développement. Nous présentons le cas d'un jeune homme qui présente un LB au niveau de la cavité buccale.

## Patient et observation

**Figure 1:**
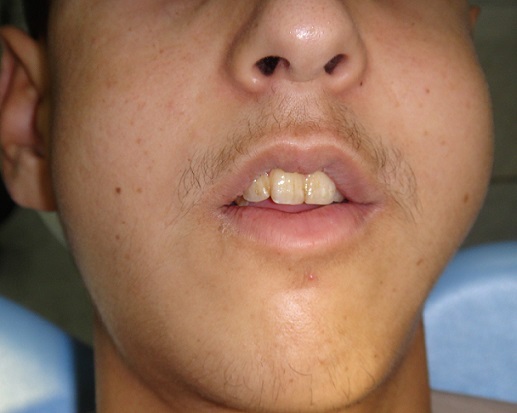
Asymétrie faciale due à la présence d’une tuméfaction génienne haute gauche

**Figure 2:**
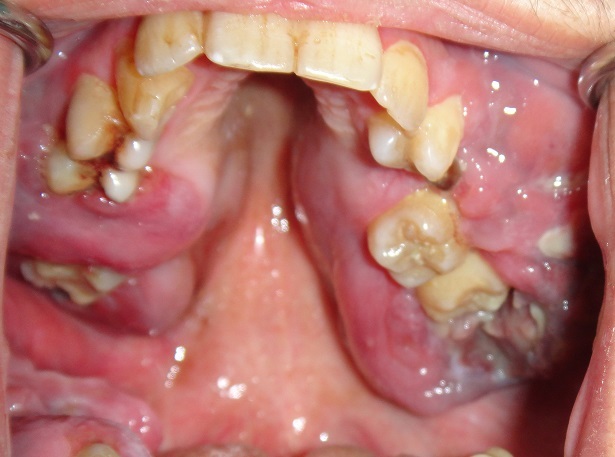
Tuméfactions endobuccales bourgeonnantes molles à la palpation avec déplacements et mobilités dentaires

**Figure 3:**
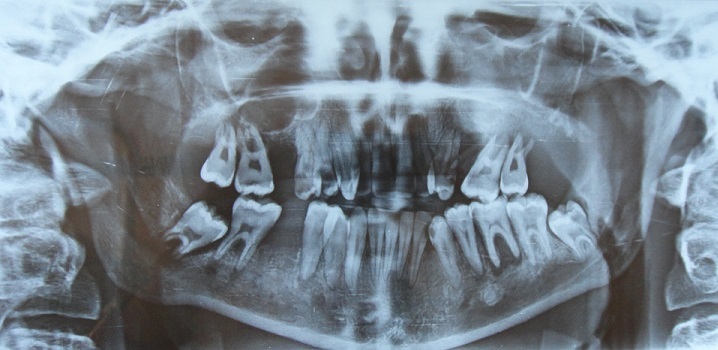
Radiographie panoramique montrant des lésions ostéolytiques avec disparition de la lamina dura et élargissement desmodontal au niveau des molaires supérieures et inférieures

**Figure 4:**
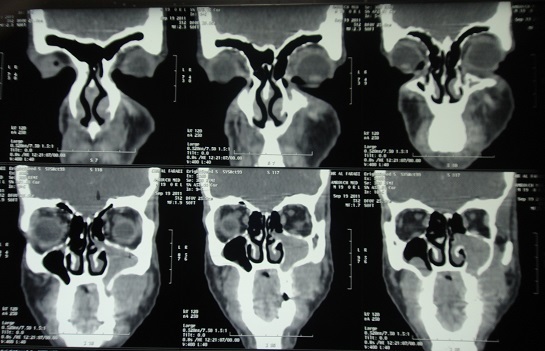
Tomodensitométrie faciale montrant un processus tumoral comblant les sinus maxillaires

**Figure 5:**
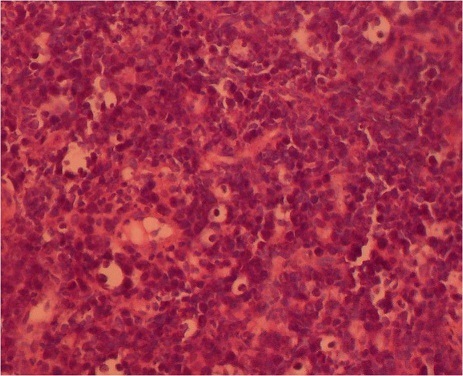
Aspect immuno-histologique : marquage pat l’anticorps CD20 confirmant le diagnostic de lymphome de Burkitt

Un jeune homme âgé de 19 ans, sans antécédents particuliers est adressé pour des tuméfactions gingivales évoluant depuis 1 mois suite à des extractions dentaires. A l'interrogatoire, le patient rapportait un état général altéré (amaigrissement, fièvre, sueurs nocturnes, prurit généralisé) ainsi que des douleurs du membre inférieur droit. Il a rapporté avoir déjà été hospitalisé pour un bilan général qui s'est avéré normal excepté une vitesse de sédimentation élevée. Par ailleurs, il n'a pas été noté de déficit moteur ni sensitif. L'examen exobuccal a révélé une asymétrie faciale gauche avec la présence d'adénopathies cervicales à la palpation des aires ganglionnaires (Figure 1). L'examen clinique endobuccal a mis en évidence la présence de tuméfactions bourgeonnantes, ulcérées par endroit plus importantes au maxillaire qu'à la mandibule recouvrant les dents avec des déplacements et mobilités. Ces tuméfactions portaient l'empreinte des dents antagonistes. A la palpation, il s'agissait de tuméfactions molles et douloureuses (Figure 2). La radiographie panoramique montrait des lésions ostéolytiques généralisées avec un élargissement desmodontal et la disparition de la lamina dura ainsi qu'une opacité comblant la moitié inférieure du sinus maxillaire gauche (Figure 3). La tomodensitométrie de la face a révélé un processus tumoral comblant les sinus maxillaires (Figure 4). A ce stade, le diagnostic évoqué était celui d'une tumeur maligne des maxillaires ou une leucémie aiguë. Une biopsie a été réalisée sous anesthésie locale dont l'examen anatomopathologique concluait à une pseudotumeur inflammatoire ulcérée comportant quelques amas atypiques nécessitant un immuno-marquage pour exclure une lésion néoplasique. L'étude immunohistochimique a été en faveur d'une localisation gingivale d'un lymphome malin non hodgkinien à grandes cellules de type Burkitt, les cellules tumorales ayant exprimé le CD20 (Figure 5).

Le patient a été adressé au service d'oncologie pour une prise en charge. Le bilan biologique a montré un taux de LDH à 931UI/l, la sérologie pour le virus de l'immunodéficience humaine (VIH) était négative. Le myologramme était normal et la cytoponction a confirmé le diagnostic du lymphome de Burkitt. Le bilan d'extension comprenant une tomodensitométrie cervico-thoraco-abdomino- pelvienne a mis en évidence une lésion intracrânienne ainsi des localisations hépatiques. Selon la classification d'Ann-Arbor, le patient a été classé au stade IV groupe C vu l'atteinte endocrânienne.

**Figure 6:**
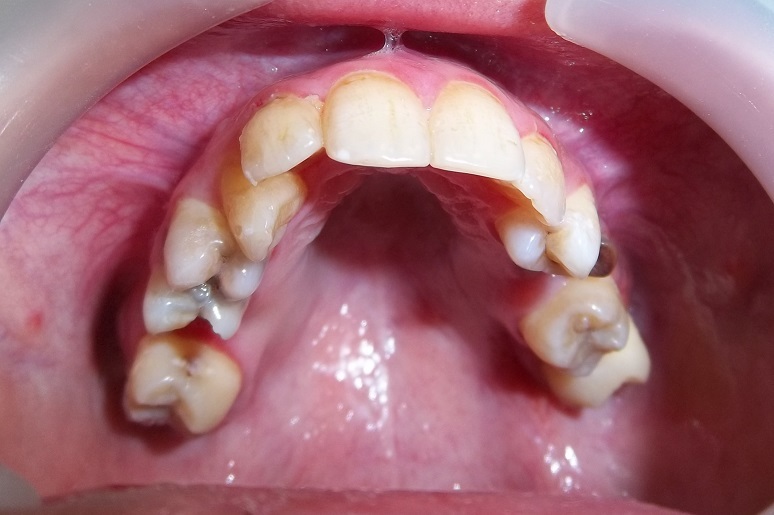
Disparition des lésions buccales après 1 mois de chimiothérapie

**Figure 7:**
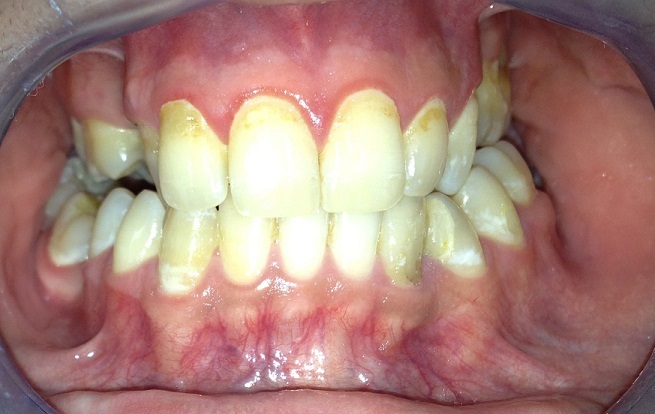
Contrôle 2 ans après fin du traitement de chimiothérapie

Le traitement a inclue 5 séances de polychimiothérapie selon le protocole LMB qui se déroule en différentes phases : une cure de COP (cyclophosphamide, vincristine, prédnisone) suivie de deux cures de COPADEM (cyclophosphamide, vincristine, doxorubicine, prédnisone) et de 2 cures de CYVE (c cytarabine, étoposide), ainsi que 9 séances de radiothérapie avec une régression totale des lésions buccales à un mois de chimiothérapie (Figure 6). Le patient a été pris en charge pour une mise en état de la cavité buccale Le contrôle à 2 ans a montré une rémission complète des lésions (Figure 7).

## Discussion

Le lymphome de Burkitt (LB) est une tumeur maligne caractérisée par la prolifération de cellules lymphoïdes de type B. Il s'agit d'un Lymphome non hodgkinien à haut grade d´agressivité avec une diffusion tumorale importante en particulier vers la moelle osseuse et le système nerveux central [4, 5].

Trois variantes cliniques ou sous types sont décrites dans la littérature [6, 7]. La forme endémique appelée lymphome de Burkitt africain. Elle est particulière par sa distribution géographique préférentielle dans les pays tropicaux. Cette haute fréquence de l'affection obéit à un mécanisme étiopathogénique regroupant d'une part l'association constante avec le virus Epstein Barr (VEB) et d'autre part la coévolution avec l'endémie majeure qu'est le paludisme à plasmodium falciparum, cofacteur favorisant l'action oncogénique du VEB et l'existence d'un dysfonctionnement immunitaire [8]. Cette forme endémique se caractérise par une prédominance de la localisation maxillaire [6]. La forme sporadique observée en occident, quant à elle, est dominée par la localisation abdominale dans 70 à 90% des cas et une moindre fréquence de l'atteinte maxillaire (10-15%) [9].

Le lymphome de Burkitt a également été décrit au cours de l'infection par le VIH. Cette forme survient à un degré d'immunodéficience avec un taux moyen de lymphocytes CD4 supérieure à 200 /mm^3^. La présentation est semblable à la forme sporadique [9]. Au Maroc, l'incidence du LB demeure inconnue. Son profil épidémiologique correspondrait d'avantage au lymphome sporadique. Dans une étude réalisée par Madani et coll en 2005, la localisation maxillaire représente 9,5% avec une atteinte abdominale dans 73,5% des cas (18) Otmani et coll en 2008 retrouvent sur 452 cas de LNH, une atteinte buccale de 8% pour le LB [10].

Ces données se rapprochent de celles rapportées dans les séries occidentales. Elles sont également rapportées par les séries du moyen orient et celles de l'Amérique du sud [3]. Ainsi, le Maroc serait une zone de transition entre la forme endémique et la forme sporadique. Plusieurs étiologies sont incriminées dans le LB : EBV, VIH, paludisme. Des études plus récentes prônent la théorie cytogénique fondée sur une anomalie chromosomique caractéristique présente dans toutes les formes de LB qu'elles soient associées ou non à l'EBV [9]. Il s'agit dans 80% des cas d'une translocation réciproque de l'oncogène c-myc du chromosome 8 vers la région des chaines lourdes des IG sur le chromosome 14. Cette translocation induit une dérégulation et la sur-expression de l'oncogène c-myc (10). Des translocations variantes t(8,22) et t(2,8) sont aussi observées respectivement dans 5à 15% des cas [7, 9]. Le LB touche essentiellement les sujets de sexe masculin avec une affinité particulière pour les os maxillaires [5]. La présentation clinique typique est une tuméfaction déformant le massif facial dont l'envahissement peut-être rapide avec une extension à tous les quadrants de la cavité buccale, au nasopharynx voire l'orbite [9].

Les caractéristiques cliniques endobuccales sont la présence de masses exophytiques de la muqueuse gingivale, de consistance ferme à la palpation associées à des déplacements et des mobilités dentaires [5, 9]. La présence d'adénopathies est un signe inconstant selon la forme du LB [4]. Une paresthésie du nerf alvéolaire inférieur peut être également retrouvée [4]. Sur le plan radiologique, des foyers d'ostéolyse avec disparition de la lamina dura et élargissement de l'espace desmodontal sont des signes précoces de la pathologie donnant aux dents un aspect flottant dans l'air [11]. Ces lésions doivent attirer l'attention du médecin dentiste. La tomodensitométrie permet d'étudier l'extension loco-régionale de la tumeur notamment au niveau osseux [11]. Le diagnostic positif est confirmé par la biopsie ou l'étude cytologique d'un frottis d'aspiration tumorale.

L'aspect histologique révèle des petites et moyennes cellules avec un noyau régulier, une chromatine réticulée immature comportant quelques nucléoles en situation souvent centrale. Il existe une importante basophilie du cytoplasme avec un aspect typique en « ciel étoilé » provoqué par la clarté des macrophages réactionnels dispersés dans une population tumorale dense et basophile [6]. L'immunophénotypage complète le diagnostic en identifiant la présence des marqueurs B. L'étude cytogénétique à la recherche d'anomalies chromosomiques et de réarrangement des gènes des Ig complète utilement l'étude histologique. Elle ne peut être effectuée que sur un fragment biopsique frais ou congelé et dans un laboratoire spécialisé [9].

Le diagnostic différentiel se fait avec un processus infectieux d'origine dentaire, avec une pathologie tumorale bénigne lorsque le lymphome de Burkitt est unilatéral. En revanche, une atteinte bilatérale suggère fortement l'origine tumorale [9]. Le lymphome de Burkitt est une urgence. La stratégie thérapeutique est parfaitement définie par des protocoles régulièrement actualisés visant à obtenir la meilleure efficacité avec une toxicité minimale [9]. La priorité est d'effectuer une évaluation rapide et complète de l'extension tumorale, critère essentiel du pronostic et du choix thérapeutique [9].

La polychimiothérapie constitue actuellement le centre du traitement du fait de la forte chimiosensibilité et de la tumeur. Le pronostic dépend du degré d'extension initiale et de la rapidité d'instauration du traitement. Plusieurs nouveaux protocoles thérapeutiques dotés d'une grande efficacité ont été développés. Les produits majeurs qui constituent la base des différents protocoles multicentriques sont le cyclophosphamide, le méthotrexate, la cytarabine, la vincristine et la doxorubicine [9]. Le taux de survie atteint 90% tous stades confondus grâce aux nouveaux protocoles LMB développés en France par la Société Francophone d'Odontologie Pédiatrique et les protocoles BFM-B développés en Allemagne [10]. Notre patient ayant bénéficié du protocole LMB est actuellement en rémission complète à 2 ans de suivi.

## Conclusion

Le lymphome de Burkitt est une tumeur maligne rare ayant une affinité pour les os maxillaires et dont l'évolution est rapide. Le médecin dentiste a un rôle dans le dépistage précoce devant le tableau clinique et radiologique caractéristique de cette tumeur ainsi que dans l'orientation des patients pour une prise en charge rapide.

## References

[1] Bouayed K, Bousfiha AA, Madani A, Zafad S, Harif M, Benchekroun S (2006). Une tuméfaction amygdalienne unilatéral de l'enfant: savoir évoquer un lymphome. Archives de pédiatrie..

[2] Satishchandra H, Sridhar AS, Pooja BP (2013). Imaging of Burkitt's lymphoma-abdominal manifestations. J Cancer Res Ther..

[3] Madani A, Benhmiddoune L, Zafad S, Harif M, Quessar A, Benchekroun S (2005). Traitement du lymphome de Burkitt de l'enfant par le protocole LMB89 à Casablanca. Bull Cancer..

[4] Patik K, Mahma VG, Jayanth B, Ambika L (2007). Burkitt's lymphoma in an Indian girl: a case report. J Indian Soc Pedod Prev Dent..

[5] Freitas R, Souza lobao veras S, Quindere LB (2008). Oral Burkitt Lymphoma: case report. Rev Bras Otorrinolaringol..

[6] Orem J, Mbide EK, Lambert B, De sanjose S, Weiderpass E (2007). Burkitt's lymphoma in Africa, a review of the epidemiology and etiology. Afr Health Sci..

[7] Kikuchi K, Inoue H, Miyazaki Y, Ide F, Matsuki E, Shigematu H, Okamoto S, Sakashita H, Kusama K (2012). Adult Sporadic Burkitt lymphoma of the oral cavity: a case report and literature review. J Oral Maxillo fac Surg..

[8] Koffi AG, N'dathz E, Tolo A, Nanho DC, Meite N, Ayemou R, Kouehion P, Sanogo I (2010). Localisations exceptionnelles du lymphome endémique de Burkitt (à propos de 21 cas vus en cote d'ivoire). Cahiers Santé..

[9] Rapp C, Simon F, Nicolas X, Jeandel P (2003). Les atteintes osseuses au cours des tumeurs endémiques viro-induites : exemples de la maladie de kaposi et du lymphome de Burkitt. Revue du rhumatisme..

[10] Otmani N, Khattab M (2008). Oral Burkitt's lymphome in children: the Moroccan experience. Int J Oral Maxillofac Surg..

[11] Lelo T, Malenga MP, Ndoma K, Bieleli E (1992). Le lymphome de Burkitt à localisation maxillo-faciale: aspects radiologiques. Médecine d'Afrique noire..

